# Supplementary material for: Heparin-network-mediated long-lasting coatings on intravascular catheters for adaptive antithrombosis and antibacterial infection
Source: Nat Commun. 2024 Jan 2;15:107. doi: 10.1038/s41467-023-44478-3 (PMC10761715; doi:10.1038/s41467-023-44478-3)
Supplement: Supplementary file 1 — Supplementary Information [file 41467_2023_44478_MOESM1_ESM.pdf]

Supplementary Information for

Heparin-Network-Mediated Long-lasting Coatings on Intravascular Catheters for Adaptive Antithrombosis and Antibacterial Infection

Lin Liu[1,2], Huan Yu[1,2], Lei Wang[1], Dongfang Zhou[3], Xiaozheng Duan[1], Xu Zhang[1], Jinghua Yin[1], Shifang Luan[1,2]*, Hengchong Shi[1,2]*

[1]State Key Laboratory of Polymer Physics and Chemistry, Changchun Institute of Applied Chemistry, Chinese Academy of Sciences, Changchun 130022, China.

[2]University of Science and Technology of China, Hefei 230026, China.

[3]NMPA Key Laboratory for Research and Evaluation of Drug Metabolism, Guangdong Provincial Key Laboratory of New Drug Screening, School of Pharmaceutical Sciences, Southern Medical University, Guangzhou 510515, China.

These authors contributed equally to this work: Lin Liu, Huan Yu.

*Corresponding author. Email: sfluan@ciac.ac.cn; shihc@ciac.ac.cn

This file includes:

Supplementary Methods
Supplementary Figures 1-20
Supplementary Table 1
Supplementary Discussion
Supplementary References

Supplementary Methods

Materials

Dimethyloctadecyl[3-(trimethoxysilyl)propyl]ammonium chloride (DAC_{18}-Cl, 60 wt.% in methanol), and stearyl trimethyl ammonium chloride (QAC-Cl) (purity = 98%) were purchased from Shanghai Aladdin Biochemical Technology Co., Ltd. N-Trimethoxysilylpropyl-N,N,N-trimethylammonium chloride solution (DAC_1-Cl, 50% in methanol) was purchased from J&K Scientific Ltd (Guangzhou, China). Tetradecyldimethyl (3-trimethoxysilylpropyl) ammonium chloride solution (DAC_{14}-Cl, 50% in methanol) was obtained from Beijing Huawei Ruike Chemical Co., Ltd. Decyldimethyl(3-trimethoxysilylpropyl) ammonium chloride solution (DAC_{10}-Cl, 50% in methanol) and pentyldimethyl(3-trimethoxysilylpropyl) ammonium chloride solution (DAC_5-Cl, 50% in methanol) were provided by Shanghai Banli Technology Co., Ltd. Heparin sodium salt (HS-Na, Poency 185 USP units/mg) was purchased from Shanghai Macklin Biochemical Co., Ltd. Dimethylsulfoxide, methanol, acetone, ethyl acetate, dichloromethane, toluene, petroleum ether concentrated sulfuric acid, calcium chloride ($CaCl_2$) and other related chemicals were all purchased from Xilong Scientific Co., Ltd (Shenzhen, China) Sterile phosphate-buffered saline (PBS, 0.01 M) was obtained from Solarbio Science & Technology Co., Ltd (Beijing, China). Kinetichrome™ Anti-IIa and Anti-Xa were purchased from Provision Kinetics, Inc (USA). BCA protein kit was purchased from Biosharp Co., Ltd (Hefei, China). Fluorescein isothiocyanate-labeled Fg (FITC-Fg), Cell Proliferation and Cytotoxicity Assay Kit (CCK-8) and Hematoxylin and Eosin Staining Kit (H&E staining) were purchased from Solarbio Technology Co., Ltd (Beijing, China). Anti-fibrinogen gamma chain antibody, mouse monoclonal [5A6] (ab119948, 1:1000), anti-CD14 antibody, rabbit monoclonal [EPR21847] (ab221678, 1:1000), anti-CD3 antibody and rabbit monoclonal [SP162] (ab135372, 1:1000) were purchased from Abcam. Alexa Fluor 555-labeled Donkey Anti-Rabbit lgG (H + L) antibody (A0453, 1:200) and Alexa Fluor 488-labeled Goat Anti-Rabbit lgG (H + L) antibody (A0423, 1:200) were purchased from Beyotime Biotechnology (Shanghai, China). L929 murine fibroblast cell line (FH0534) was purchased from FuHeng Biology (Shanghai, China). TNF alpha Mouse Uncoated ELISA Kit with Plates (Catlog # 88-7324-22) and IL-6 Mouse Uncoated ELISA Kit with Plates (Catlog # 88-7064-22) were purchased from Thermo Fisher Scientific Inc (Shanghai, China).

Synthesis and characterization of complexes

Preparation of complexes

Several oppositely charged DAC_X-Cl (X referred to the different alkyl chain lengths, X = 1, 5, 10, 14, 18) and HS-Na were used to prepare complexes. Briefly, DAC_X-Cl with different molarity (ranging from 10^{-7} to 4×10^{-5} mM) and HS-Na (0.02 g/mL, chose the mass concentration due to the HS-Na is a mixture) were dissolved in deionized water. After that, DAC_X-Cl solution was added dropwise into the HS-Na solution with continuous stirring. The resulting precipitate was thoroughly rinsed with deionized water until no precipitation occurred upon adding silver nitrate solution to the supernatant. Finally, the precipitate was subjected to freeze-drying.

Characterization of HS/DAC complexes

DAC an alkyl chain length of 18 was selected based on the amount of precipitation during the complex formation process. The HS/DAC complex with the highest yield was obtained from the reaction between DAC-Cl (4×10^{-5} mM) and HS-Na (0.02 g/mL) and underwent further characterization. This included analyses using attenuated total reflectance Fourier transform infrared spectroscopy (ATR-FTIR, BRUKER Vertex 70) and X-ray Photoelectron Spectroscopy (XPS, VG Scientific ESCA MK II Thermo Avantage V 3.20 analyser).

Surface characterization of the HS/DAC-coated samples

To examine the surface morphology and coating thickness of HS/DAC-coated samples, Atomic Force Microscopy (AFM, Seiko, Japan) and Model XL 30 ESEM FEG (SEM, Philips, Netherlands) were employed, respectively. AFM utilized a spring constant of approximately 2 N/m, and 30 μm image scans. The static water contact angles of HS/DAC-coated samples were determined using a contact angle goniometer drop-shape analysis (KRÜSS GMBH, Germany)[1]. The chemical structure and composition of HS/DAC-coated samples were further analyzed by ATR-FTIR and XPS. UV-Visible Spectrophotometer (Lambda 35, PerkinElmer, USA), ranging from 200 to 800 nm, was employed to evaluate the transmittance of HS/DAC-coated TPU.

Substrate-independence of HS/DAC coating

The substrate independence of the HS/DAC coating was verified by applying the coating to plain glass tubes and various medical devices made of polymer materials. For polymer medical device materials, a hydroxylated surface was achieved through 5 min of plasma treatment. As for plain glass tubes, the modified surface was obtained by boiling them in a piranha solution for 1 h. Subsequently, the hydroxylated material was soaked in a dichloromethane solution of HS/DAC (10 mg/mL) for 1 min. The samples were air-dried, washed 3 times with deionized water and cured at 100 °C. Finally, the coated samples were sonicated in deionized water for 5 min and washed 3

times and dried at room temperature. HS/DAC-coated devices were stained with Rose Bengal for further observation.

Antithrombogenic Properties of HS/DAC-coated substrates

Anticoagulant activity of the HS/DAC coating

The anticoagulant activity of (HS/DAC)-TPU was assessed using chromogenic anti-FXa assay (Kinatichrome^{TM} Heparin Anti-Xa Kit) and anti-FIIa assay (Kinatichrome^{TM} Heparin Anti-IIa Kit). *Chromogenic anti-FXa assay:* Bare TPU films and (HS/DAC)-TPU films were incubated in PBS for 2 min at 37 °C. Subsequently, antithrombin solution was added, followed by FXa solution and a substrate solution of Xa (CH_3OCO-D-CHA-Gly-Arg-pNA-AcOH). The conditions for each incubation were 37 °C for 2 min. The reaction was stopped with acetic acid (20 v/v%), and the absorbance was measured at 405 nm using a microplate spectrophotometer (SYNERGY). A blank solution was prepared by adding each assay solution in reverse order. Similar to the FXa assay, bare TPU films and HS/DAC-coated TPU films were incubated in PBS (37 °C, 2 min). The subsequent addition of antithrombin, thrombin, a substrate solution of IIa (H-D-Phe-Arg-pNA•2HCl), and acetic acid concluded the reaction. Absorbance was recorded at 405 nm. A blank solution was also created by adding each assay solution in reverse order.

BSA adhesion assay

Samples were soaked in PBS solution of Bovine Serum Albumin (BSA, 1 mg/mL) for 1 h at 37 °C, followed by PBS washing. The BCA protein kit was used to detect the amount of protein adsorbed on the sample surface. Sodium lauryl sulfate solution (0.07 M) was added to each sample, shaken for 2 h to detach surface proteins, and supplemented with an equal amount of BCA reagent. After incubation at 60 °C for 1 h, absorbance was measured at 560 nm (SYNERGY).

Fibrinogen adsorption and activation

An in vitro circulation system was used to determine Fg adsorption and activation on surfaces. First, fresh blood from rats was drawn into vacuum tubes containing sodium citrate (volume ratio of anticoagulant to blood = 1:9). Both bare and HS/DAC-coated catheters made of polyurethane (TPU) were cut into 10 cm pieces. All catheters had been previously sterilized and rinsed with saline solution thoroughly. Thereafter, these catheters were implanted into silicone rubber catheters with an inner diameter of 3 mm and outer diameter of 5 mm, forming a closed loop system with 3 mL recalcified blood ($CaCl_2$ saline solution, 0.045 M, 70 μL/mL) containing fluorescein isothiocyanate-labeled Fg (FITC-Fg, 5 v/v%). After 2 h of flow (1 mL/min), samples

were gently rinsed with PBS, fixed in 4% paraformaldehyde, and observed under confocal laser scanning microscopy (CLSM, LSM 700, Carl Zeiss). Fg conformational changes expose γ chain, which then bind to GPIIb/IIIa on the platelet membrane, resulting in platelet aggregation. Therefore, the activation of Fg was detected with the immunofluorescence staining method. Except that no FITC-Fg was added, the process was similar to the above. After fixing with 4% of paraformaldehyde, samples were cleaned with PBS. Next, samples were immersed in monoclonal rabbit anti-mouse fibrinogen γ chain antibody at 37 °C for 1 h and rinsed with PBS 3 times. Subsequently, Alexa Fluor 555-labeled Donkey Anti-Rabbit lgG (H + L) antibody was added to the samples and incubated for 1.5 h at 37 °C. Finally, the samples were rinsed with PBS 3 times and freeze-dried to observe by CLSM.

Adhesion of Inflammatory Cells

The circulation system mentioned above was also utilized to test the adhesion of inflammatory cells on catheters. Briefly, samples were circulated in fresh whole blood from mice, as described above. Samples were removed and gently rinsed with PBS after 2 h through the closed circular tube loop. For monocyte and lymphocyte adhesion test, catheter segments (0.5 cm) were fixed in 4% of paraformaldehyde and rinsed with PBS. These segments were incubated in primary antibodies against monocytes (anti-CD14) and lymphocytes (anti-CD3) for 1 h. Samples were rinsed with PBS and further incubated for 1.5 h in fluorescently conjugated secondary antibodies (lgG (H + L), Alexa Fluor 488-labeled Goat Anti-Rabbit). Finally, stained tubes were rinsed and freeze-dried and observed by CLSM. For the neutrophil adhesion test, 4',6-diamidino-2-phenylindole (DAPI) with 405 nm excitation was used to label the cell nucleus. Catheter segments were fixed in Triton X-100 (diluted 1:1000 in PBS) for 10 min and cleaned with PBS for 3 times. Next, samples were immersed in DAPI (10 μg/mL) solution for 10 min and rinsed with PBS. Finally, samples were observed by CLSM.

Clotting time and anticoagulation property of HS/DAC coating in vitro

Fresh blood from rats was drawn into vacuum tubes containing sodium citrate. The blood (2 mL) was added to bare glass bottles and HS/DAC-coated bottles and recalcified using $CaCl_2$ in saline (0.045 M, 140 µL). The tube was inverted until no blood flows. Coagulation time and coagulation status were recorded. A commercial infection-resistant CVC catheter (Arrowg + ard Blue, 16 Ga, Teleflex) was selected as control sample to compare the antithrombotic properties with HS/DAC-coated CVC catheter. Blood from rabbit was drawn into vacuum tubes containing sodium citrate

(volume ratio of anticoagulant to blood = 1:12). CVC, (Arrowg + ard Blue)-CVC, and (HS/DAC)-CVC were cut into 3 cm pieces. All catheters had been previously sterilized and rinsed with saline solution thoroughly. Samples were respectively implanted into silicone rubber catheters with an inner diameter of 3 mm and connected to a peristaltic pump (LEAD FLUID, BT101 L). Then, the blood (3 mL) was recalcified using $CaCl_2$ in saline (0.045 M, 150 μL) and injected into the peristaltic pump to form a closed loop system, and incubated at 1 mL/min for 2 h. Samples were gently rinsed with PBS and weighed.

Ex vivo evaluation of thrombogenicity

Bare catheters, HS/DAC-coated catheters, heparinized polyvinyl chloride (PVC) catheters and surgical devices were used in ex vivo. The extracorporeal circulation was established as an arteriovenous shunt model following the reported procedure.[2, 3]. Three adult New Zealand white rabbits (3.0-3.5 kg) were general anesthetized. The left carotid artery and the right external jugular vein of rabbits were isolated, and they were connected to build the extracorporeal circuit. Both the bare and HS/DAC-coated catheters were assembled into the rabbit arteriovenous shunt. The samples were photographed and weighed after 2 h of treatment.

Thrombogenicity studies in canine in vivo

Beagles (n = 3, 12-14 kg) were anesthetized with propofol and the isoflurane inhalation was used throughout the surgical procedure. Commercial CVCs and HS/DAC-coated CVCs were inserted approximately 20 cm into the right and left jugular veins of canines. After implantation for 24 h, canines were anesthetized by propofol and euthanized by intravenous injection of 10% potassium chloride. Jugular veins with CVCs were removed to examine thrombi formation. Veins were opened lengthwise and underwent H&E staining to test the biocompatibility of HS/DAC-coated CVCs in veins.

Anti-coagulant stability of coating in salt solution

HS/QAC-coated TPU and HS/DAC-coated TPU were immersed into 0.9% NaCl solution with sharking (100 rpm) at 37 °C. Samples were removed on different days (0, 10, 20, 30 days) and rinsed with deionized water. Then, the anticoagulant ability of the samples was determined by chromogenic anti-FXa assay and anti-FIIa assay described above.

Antithrombotic stability of coating in flowing artificial blood

HS/DAC-coated catheters with inner diameter of 0.3 cm were connect to the silicone tube. A peristaltic pump was utilized to connect the channel. Artificial blood was injected into the

peristaltic pump to form a closed loop system. The antithrombotic stability of coated catheters after 30 days with flow rates of 4.5 and 45 mL/min was investigated respectively. The nature of blood flow was represented by a power law fluid[4, 5]. The shear rate of a non-Newtonian fluid can be calculated by the following formula, where n is the power-law index, Q is the flow rate, and R is the diameter of the catheter.

$$\gamma = \frac{3n+1}{n} \times \frac{8Q}{\pi R^3} \qquad (1)$$

Therefore, flow rates of 4.5 and 45 mL/min correspond to shear rates of 30 s^{-1} and 300 s^{-1} respectively. The peristaltic pump was cycled every 12 h to prevent severe heating. Bare catheter and HS/DAC-coated catheter were cut into 3 cm pieces after 30 days. Fresh blood from rabbit was drawn into vacuum tubes containing sodium citrate (volume ratio of anticoagulant to blood = 2:8). Samples were connected to the silicone tube. After that, the blood was recalcified by using of $CaCl_2$ in saline (0.045 M, 150 µL/mL) and injected into the peristaltic pump to form a closed loop system, and incubated at 1 mL/min for 1 h. Samples were gently rinsed with PBS and used for weighing.

Antithrombotic stability of coating in vivo

Coated or uncoated TPU films with a diameter of 0.8 cm were implanted into the subcutaneous tissue on both sides of the back of rats. After 10 and 30 days of implantation, samples were taken out and rinsed. The fresh blood from rabbit was calcified using $CaCl_2$ in saline (0.045 M, 100 µL). Bare TPU films and HS/DAC-coated TPU films at day 0, day 10 and day 30 were immersed in blood (200 µL). and the sample was observed for surface thrombosis and weighed after 1 h.

Antibacterial ability of HS/DAC-coated substrates

Antibacterial types of HS/DAC

The inhibition zone test was employed to investigate the antibacterial types of HS/DAC. Staphylococcus aureus (*S. aureus*, ATCC 6538) was obtained as described previously[1]. In brief, *S. aureus* strains from -80 °C frozen stocks were streaked onto a lysogeny broth (LB) agar plate. The plate was then incubated overnight at 37 °C until individual colonies were visibly formed. The single colony was selected and transferred to LB medium. The inoculated medium was incubated overnight at 37 °C under 110 rpm until it reached the exponential growth phase. Dilute the suspension in fresh LB medium to obtain a bacterial solution with an optical density of 0.01 at 10^6 CFU/mL. Bacterial suspensions (200 µL, 10^6 CFU/mL) were dropped onto agar plates and

inoculated evenly. Then, the TPU wafers with 0.5 cm diameter were placed on agar plates and incubated at 37 °C for 24 h.

Broad-spectrum antibacterial activity of films

The broad-spectrum antibacterial properties of HS/DAC-coated substrates were assessed using *S. aureus* and *Escherichia coli* (*E. coli*, ATCC 25922) as representatives of gram-positive and gram-negative bacteria. Sterilized TPU films (1.5 × 1.5 cm^2) underwent colony count analysis[1], where bacteria (25 μL, 10^6 CFU/mL) were dropped onto the surface, covered with PE film (1 × 1 cm^2), and incubated for 12 h. After incubation, PBS was added for ultrasound and colony counting was conducted. Additionally, bare TPU films and HS/DAC-coated TPU films (1 × 1 cm^2) were immersed in bacterial suspension (10^6 CFU/mL) and incubated with bacteria for 24 h. Samples were rinsed with PBS and fixed in 4% of paraformaldehyde. A portion of the bare TPU films and HS/DAC-coated TPU films samples were freeze-dried for imaging under SEM, and other samples were stained with a LIVE/DEAD BacLight Bacterial Viability Kit (Molecular Probe Inc., USA) and observed by CLSM[6].

Anti-biofilm activity of films

TPU and HS/DAC-coated TPU (1.0 cm × 0.8 cm) were incubated in LB (200 μL) containing 10^6 CFU/mL of *S. aureus*. After the incubation at 37 °C for 12 h, surfaces were gently rinsed with PBS and fresh LB medium was added. Colony plating was performed after 48 h. Then SEM was used to observe the biofilm. Samples were also fixed with 4% polyformaldehyde for 6 h and observed with SEM to evaluate biofilm formation.

Antibacterial property of HS/DAC-coated catheters

The antibacterial properties of HS/DAC-coated catheters were tested through colony count analysis in both static and flow environments, as described previously[7]. Briefly, bacterial cultures (200 μL, 10^6 CFU/mL) were injected into lumen of catheters and incubated at 37 °C for 3 h. Subsequently, samples were rinsed with PBS for 3 times, injected with fresh LB (200 μL), and sonicated for 5 min after 24 h. Bacterial cultures were serially diluted with PBS for colony counting. For the antibacterial activity of HS/DAC-coated catheters in a flowing environment, the catheter segments (4 cm) were incubated in bacterial suspension (10^6 CFU/mL) at 37 °C for 3 h. The bacterial suspension was removed and the catheters were gently rinsed with PBS. Subsequently, a peristaltic pump was utilized to connect the catheters. Catheters were incubated in fresh LB medium at a flow rate of 0.1 mL/min. Samples were obtained at 24 h and 48 h, and the colony

count of bacteria adhered to catheters was conducted. We also investigated the antimicrobial properties of (HS/DAC)-coated catheters versus commercial antibacterial catheters. Arrowg + ard Blue catheter (16 Ga, Teleflex) was selected as a commercial infection-resistant CVC catheter in this assay. Bare CVC, (Arrowg + ard Blue)-CVC and (HS/DAC)-CVC were immersed in the bacterial solution (1 mL, 10^6 CFU/mL) for 12 h. Samples were lightly rinsed with PBS and added with 6 mL PBS solution for ultrasound, and then 200 μL of solution were taken for colony count.

Antibacterial properties of HS/DAC-coated catheters in vivo

Nine female BALB/C mice at 6-8 weeks were used to construct the subcutaneous model of indwelling catheter-related infection[8]. Mice were housed at an ambient temperature of 25 °C (24-26 °C), humidity of 30 % and a photoperiod of 12 h light/12 h dark. In addition, a standard diet and water were given. First, sections of indwelling catheter tips (0.4 cm) were cut off and cleaned with ethanol. Bare and HS/DAC-coated tips were disinfected by immersing in 75% ethanol for 30 min. Next, samples were incubated with *S. aureus* (10^6 CFU/mL) at 37 °C for 3 h and rinsed gently with PBS. Two incisions approximately 0.5 cm in length were made on both sides of the back of each mouse. Bare and HS/DAC-coated catheters with bacteria were implanted into the incision of the same mouse, and then the wounds were stitched. The mice were euthanized after 5 days, and the implant places were photographed. Moreover, the implanted slides and associated peri-implant tissue were surgically removed for further experiments. Three groups of implanted slides and surrounding tissues were broken into pieces, immersed in 3 mL PBS and ultrasonicated for 5 min. The number of live bacteria in tissues and catheters was counted through the colony count method. The other three groups of tissues were fixed with 4% paraformaldehyde and underwent H&E staining[8]. The remaining three groups of tissues were frozen at -80 °C and then homogenized in PBS (0.1 g/mL). The supernatant was collected to measure inflammation after centrifugation. Tumor necrosis factor-α (TNF-α) and interleukin-6 (IL-6) were chosen as cytokines to evaluate the level of inflammation. A TNF alpha Mouse Uncoated ELISA Kit with Plates and an IL-6 Mouse Uncoated ELISA Kit with Plates were utilized to measure the concentrations of TNF-α and IL-6, respectively.

Antibacterial stability of coating in salt solution

HS/QAC-coated catheters and HS/DAC-coated catheters were immersed in 0.9% NaCl with sharking (100 rpm) at 37 °C. After removal on different days (0, 10, 20, and 30 days), bacterial suspension was injected, and samples were cultivated as described in the section of antibacterial

property of HS/DAC-coated catheters in static environments. The colony-count assay was conducted to determine the activity of bacteria on the samples.

Antibacterial stability of coating in flowing artificial blood

As described in the section of the antithrombotic stability of coating in flowing artificial blood, artificial blood was used to investigate the antibacterial stability of coated catheters at flow rates of 4.5 and 45 mL/min, respectively. The HS/DAC-coated catheters at 30 days were soaked in the bacterial solution (10^6 CFU/mL) for 12 h. Samples were lightly rinsed with PBS and added with 8 mL PBS solution for ultrasound, and then 200 µL of solution were taken for colony counting.

Antibacterial stability of coating in vivo

Bare and (HS/DAC)-coated TPU films, with a diameter of 0.8 cm were surgically implanted into the subcutaneous tissue on both sides of the back of rats. After 10 and 30 days of implantations, samples were taken out and rinsed. Bacterial solution (10^6 CFU/mL) were co-cultured with the samples for 12 h. Following co-culture, the samples underwent a gentle rinse with PBS and were subsequently treated with a 4 mL PBS solution for ultrasound. 200 µL of solution were taken for colony counting.

Biocompatibility of HS/DAC coating

The biocompatibility of (HS/DAC)-TPU was evaluated by cytotoxicity assay and host response to subcutaneous implantation. Cells were prepared as previously described[1]. L929 murine fibroblast cell lines were incubated using high-glucose dulbecco's modified eagle medium (DMEM) containing 10 vol% fetal bovine serum and 1 vol% penicillin-streptomycin solution. EDTA-trypsin digestion was used and subculture to obtain stable cells. Finally, the required cell suspension was obtained by cell counting for the experiment. The cells were passed twice to obtain stable cells. Finally, the required cell suspension was obtained through trypsinization, centrifugation and cell counting for the experiment. The cytocompatibility was investigated by extraction method and contact method respectively. In the extraction method, Bare TPU, (HS/QAC)-TPU and (HS/DAC)-TPU were immersed in DMEM containing bovine serum (1%) for 24 h. The resulting extracts were co-incubated with seeded cells (1×10^4 cells/well) in 96-well plates for 24 h. CCK-8 was added and placed for 2 h to test the absorbance at 450 nm. The negative control was cells without extract and the positive control is a blank sample without cells. In the contact method, Bare TPU, (HS/QAC)-TPU and (HS/DAC)-TPU were incubated with the seeded cells (2.5×10^5 cells/well) in 24-well plates for 24 h, respectively. CCK-8 solution was then added for testing as

described above. The preparation of negative and positive control samples was the same as above. The calculation of cell viability follows the following equation:

$$Cell\ viability\ (\%) = \frac{A_{Sample} - A_{Positive}}{A_{Negative} - A_{Positive}} \times 100\% \qquad (2)$$

For the biocompatibility of the HS/DAC coating in vivo, the bare catheter and the HS/DAC-coated catheter were sterilized and implanted on both sides of the back of the mouse, similar to the above experimental procedure. The implanted slides and surrounding tissues were collected for H&E staining, and the levels of TNF-α and IL-6 were measured. Meanwhile, the venous vessels implanted with the catheter site from the canine model were also subjected to H&E staining to measure the biocompatibility of HS/DAC-coated CVCs.

Supplementary Figures

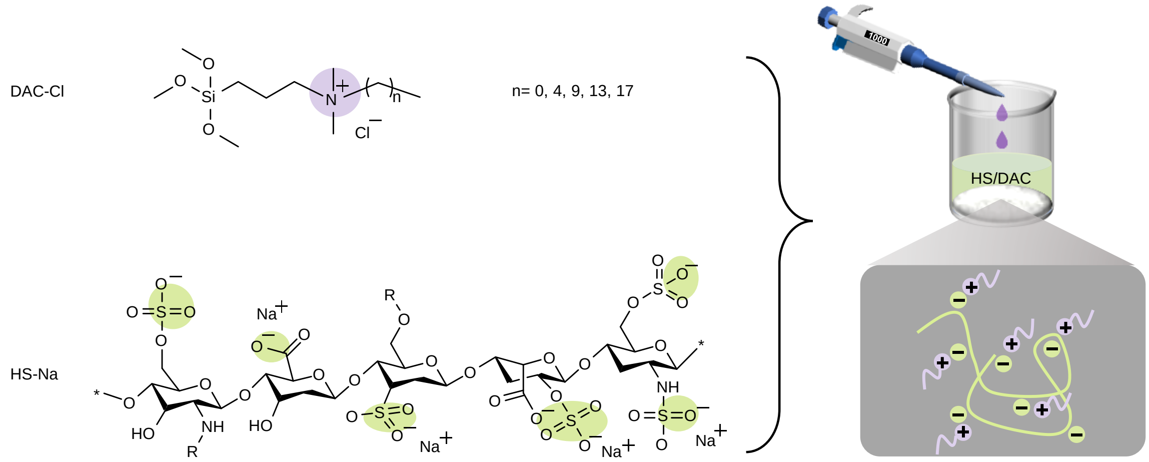

Supplementary Fig. 1 Schematic of the preparation of the multifunctional coating complexes.

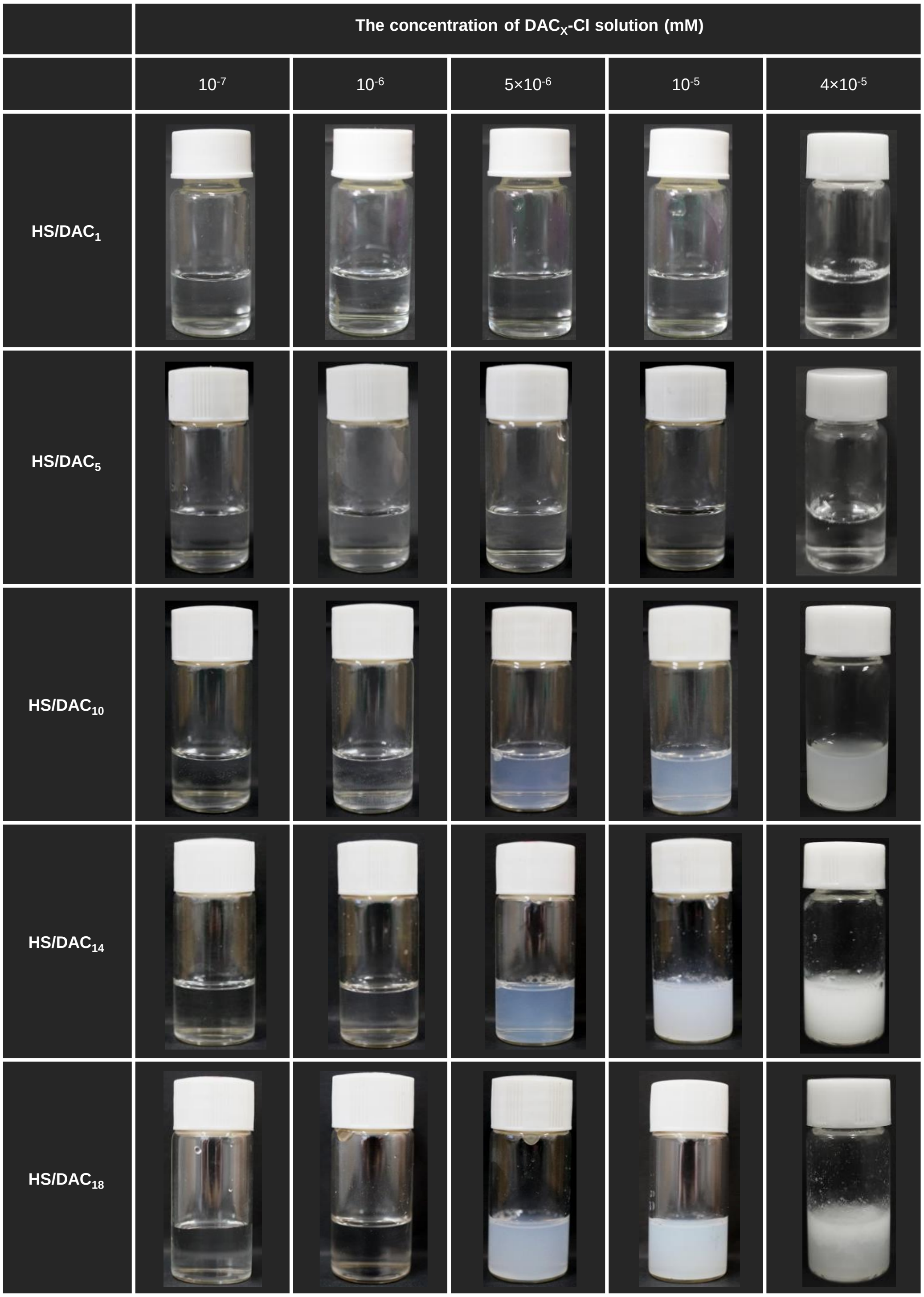

Supplementary Fig. 2 Images of the complexes formed by various ratios of HS and DAC_X. Measurements were repeated three times independently with similar results.

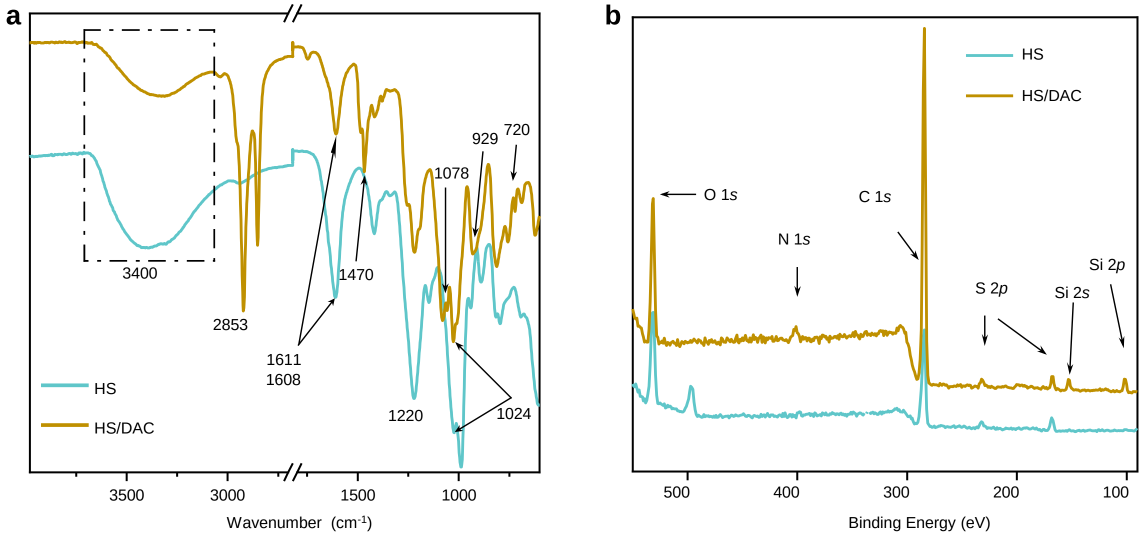

Supplementary Fig. 3 Chemical structure of the HS/DAC complex measured with FTIR (a) and XPS (b).

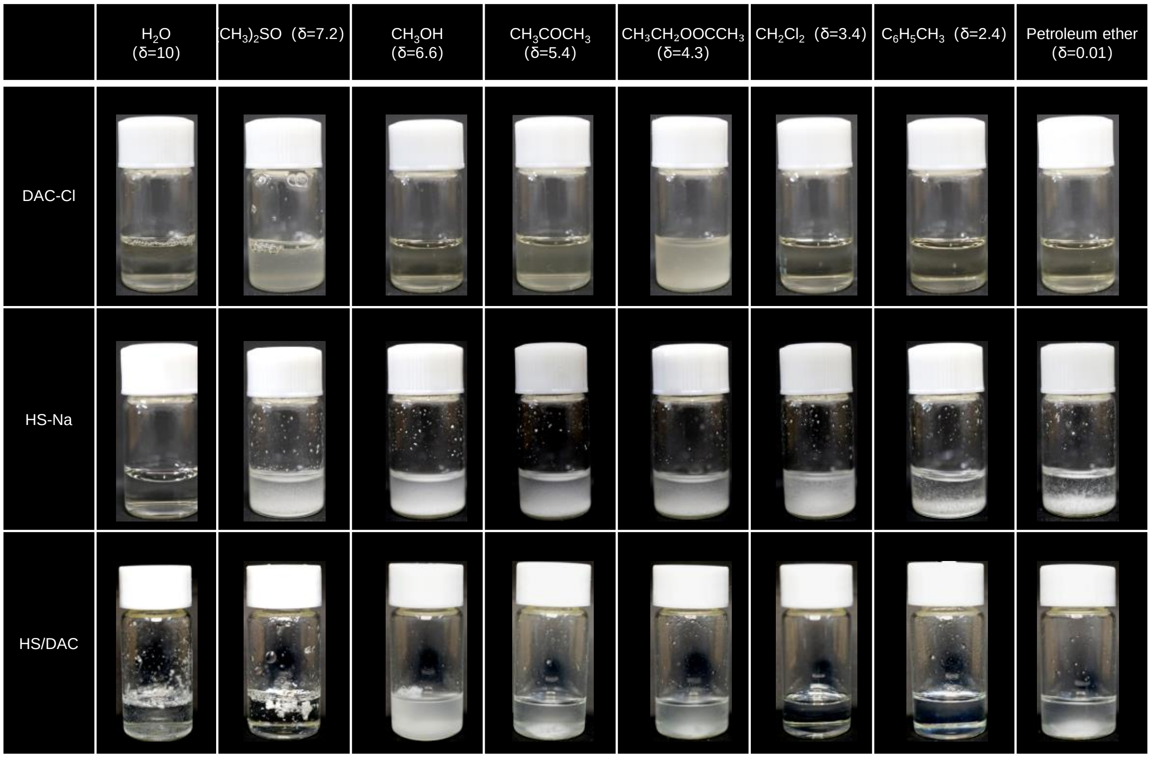

Supplementary Fig. 4 Solubility of HS/DAC complexes in different solvents. Measurements were repeated three times independently with similar results.

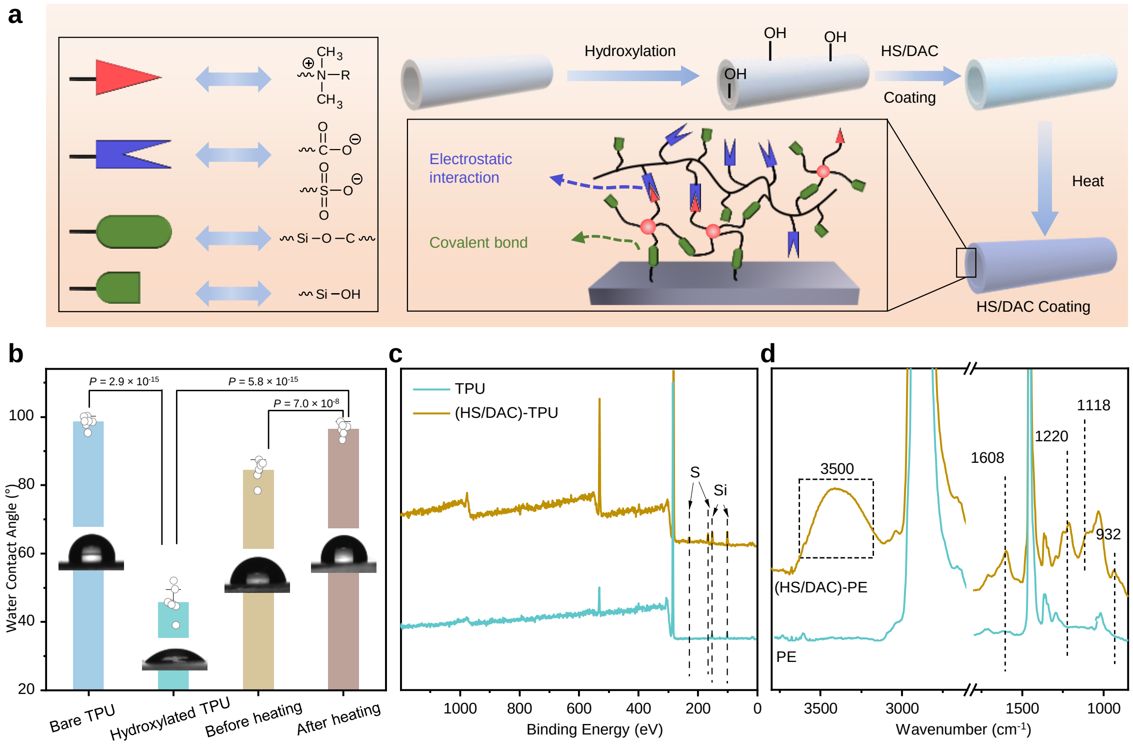

Supplementary Fig. 5 (a) Schematic illustration of the preparation of HS/DAC-coated substrates. (b) Water contact angle of TPU films in different periods during the coating formation process (mean ± SD, n = 8 independent samples). (c) XPS of TPU films with or without HS/DAC coating. (d) FTIR of PE films with or without HS/DAC coating. One-way ANOVA with Tukey's post hoc test was used in b.

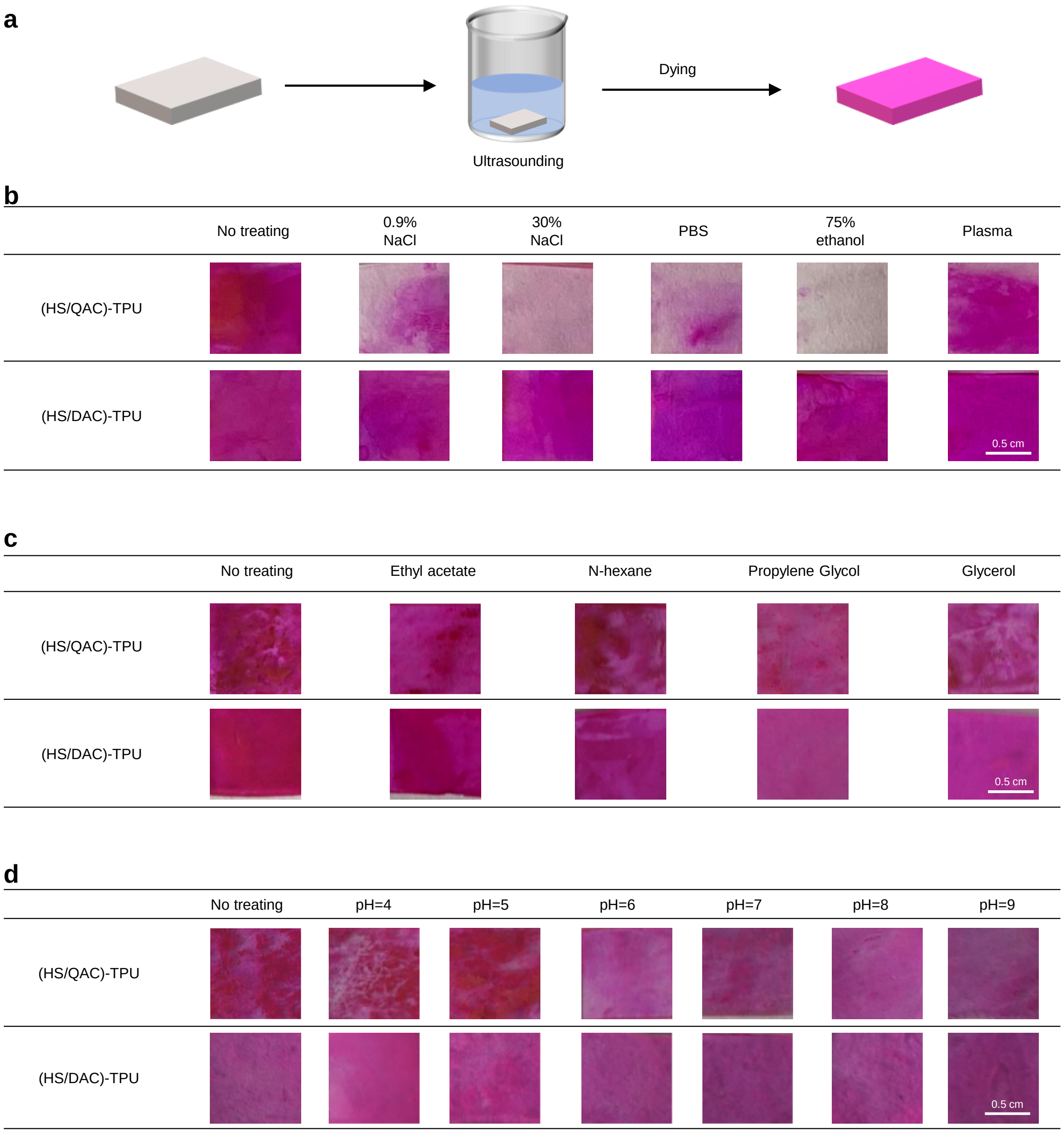

Supplementary Fig. 6 Durability of coating in solvents. (a) Schematics of the measurement of the stability of the coating in solvents. Images of (HS/QAC)-TPU and (HS/DAC)-TPU after treatment in common solvents (b), organic solvents (c) and solutions with different pH values (d). Coatings were colorized by Rose Bengal for visualization. Measurements were repeated three times independently with similar results.

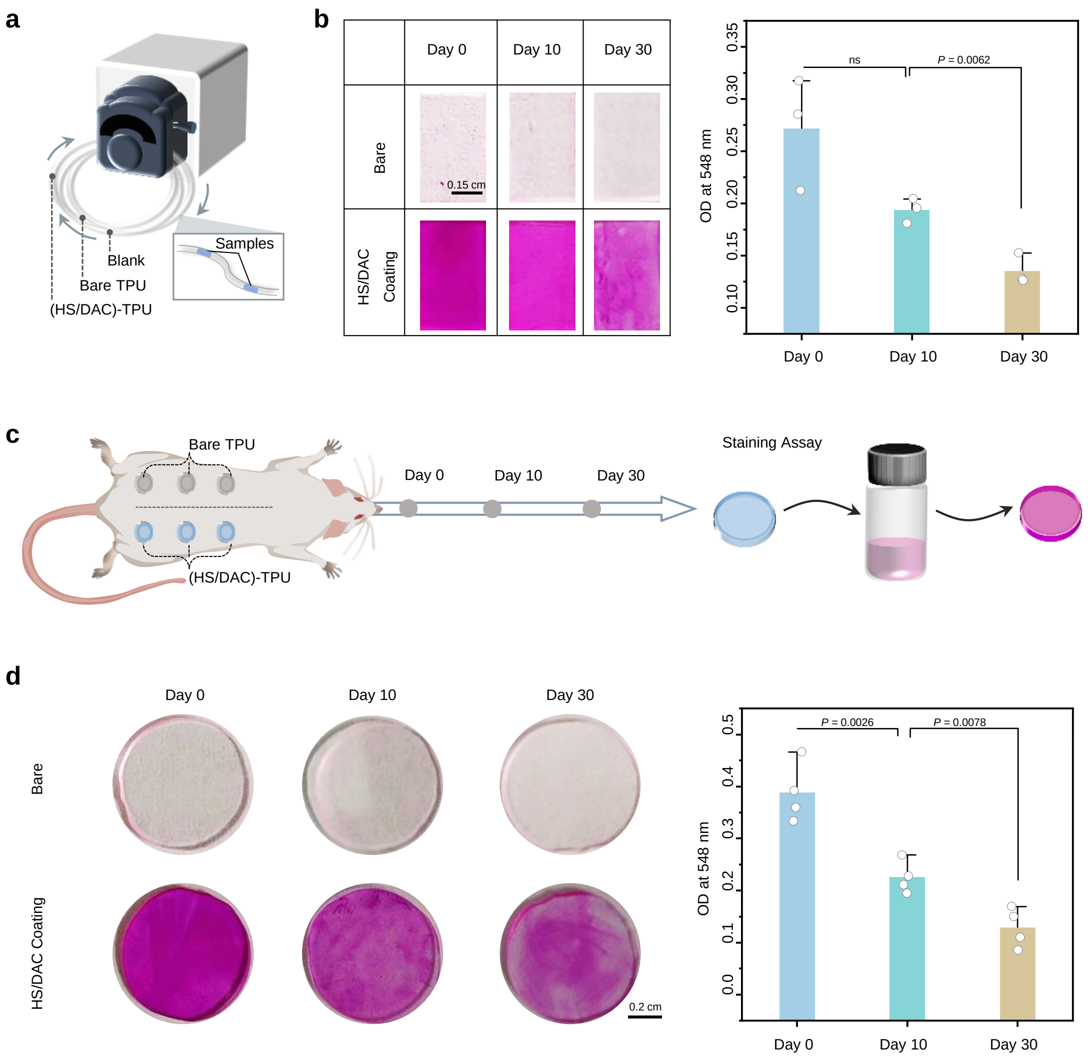

Supplementary Fig. 7 The long-term ability of HS/DAC coating. (a) The multi-channel circulation model of long-term stability with flow incubation. (b) The digital photos of the coating marked by the dye after flowing in 0.9% NaCl for 0, 10 and 30 days and the corresponding change of the absorbance intensity of the dye (mean ± SD, n = 3 independent samples). (c) The subcutaneous implantation model in rats for long-term stability. (d) The digital photos of the coating marked by the dye and the change of the absorbance intensity of the dye after implanted in vivo for 0, 10 and 30 days (mean ± SD, n = 4 independent samples). One-way ANOVA with Tukey's post hoc test was used in (b, d). ns: no significant difference. P = 0.07 in b.

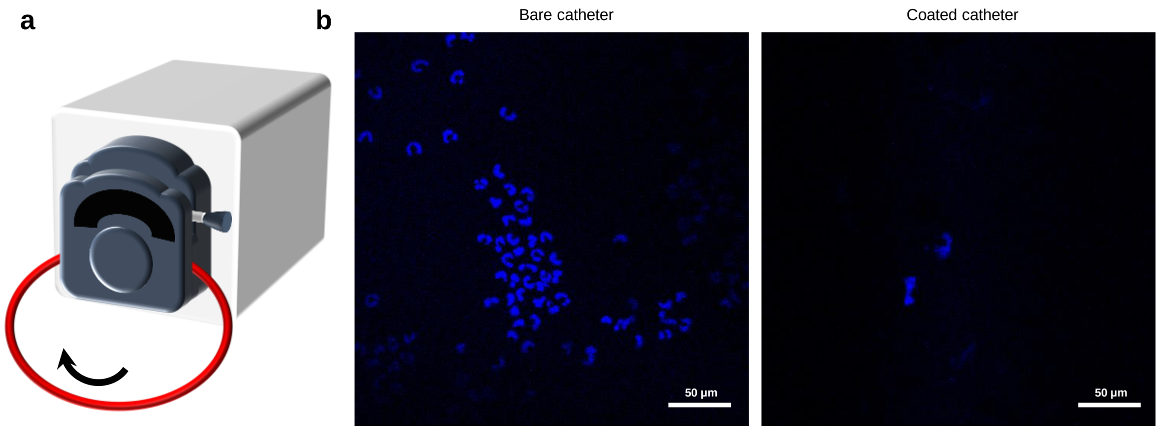

Supplementary Fig. 8 (a) The apparatus used for blood circulation test. (b) CLSM images of neutrophils adhesion on the surface of bare catheters and HS/DAC-coated catheters. Measurements were repeated three times independently with similar results.

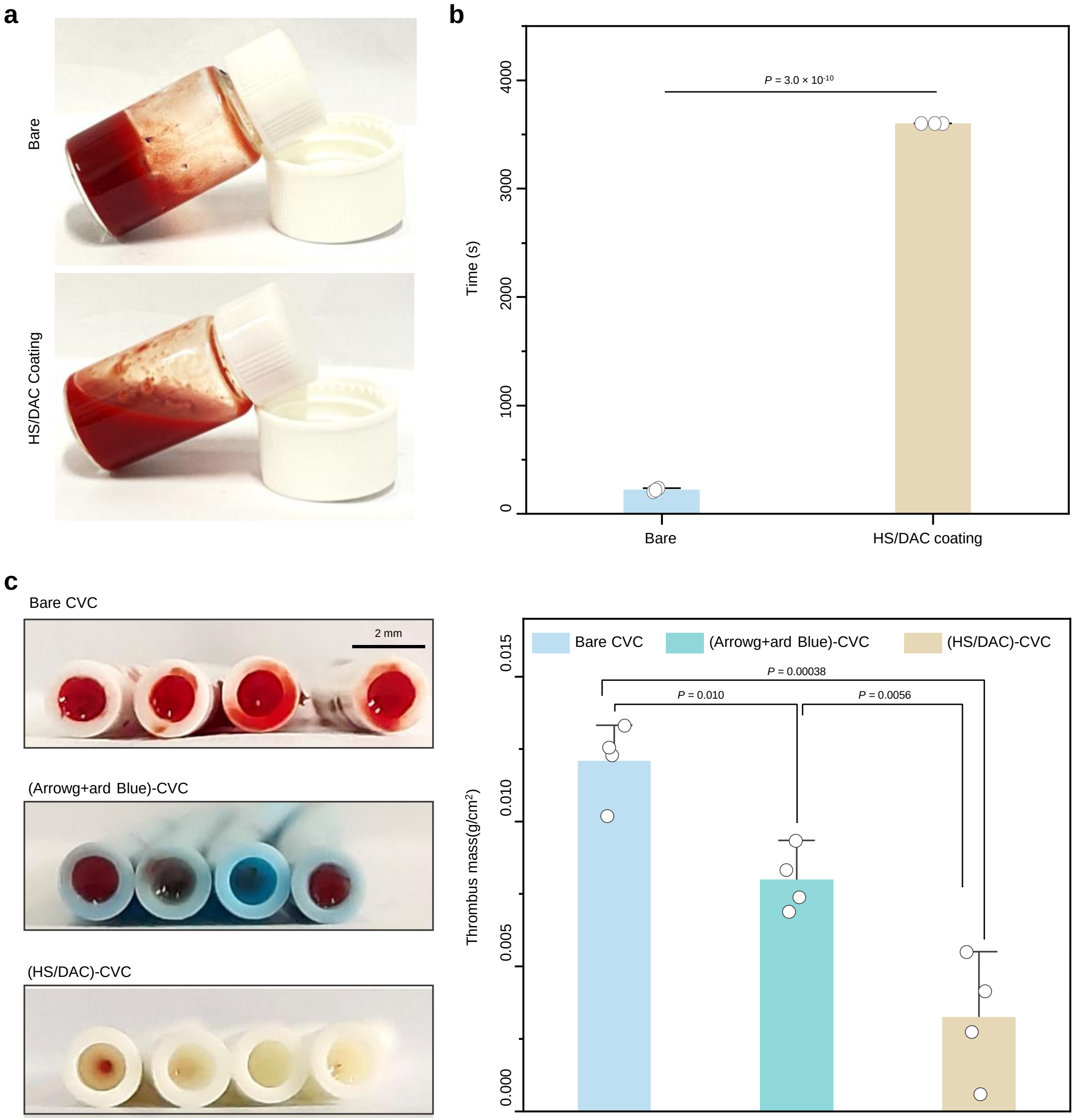

Supplementary Fig. 9 (a) Optical images of blood in HS/DAC-coated and uncoated glass bottles. Measurements were repeated three times independently with similar results. (b) Clotting-time test of blood in HS/DAC-coated glass bottles and uncoated glass bottles (mean ± SD, n = 3 independent samples). (c) Digital photos and quality of thrombi in the lumen of commercially available (Arrowg + ard Blue)-CVC and (HS/DAC)-CVC (mean ± SD, n = 4 independent samples). Two-sample Student's t test was used in b. One-way ANOVA with Tukey's post hoc test was used in c.

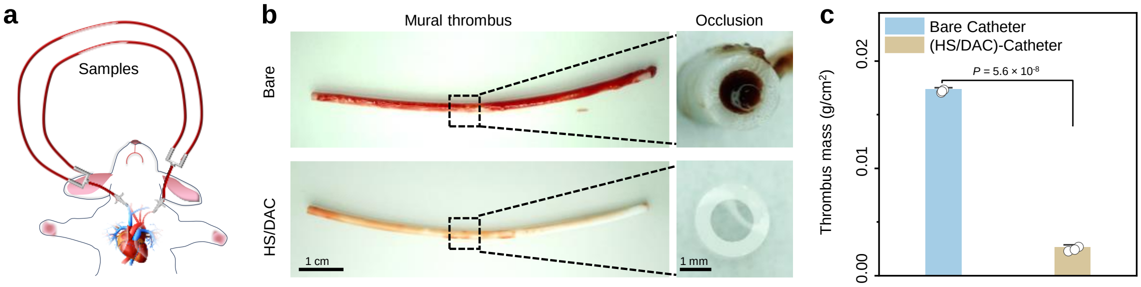

Supplementary Fig. 10 Ex vivo evaluation of the antithrombogenic properties of the HS/DAC coating in an arteriovenous shunt model. (a) Schematic diagram of the experimental design. (b) Photographs of the thrombi on the surface of samples and cross-sectional observation of the samples. Measurements were repeated three times independently with similar results. (c) Quality of thrombus formation on the surface of the catheter (mean ± SD, n = 3 independent samples). Two-sample Student’s t test was used in c.

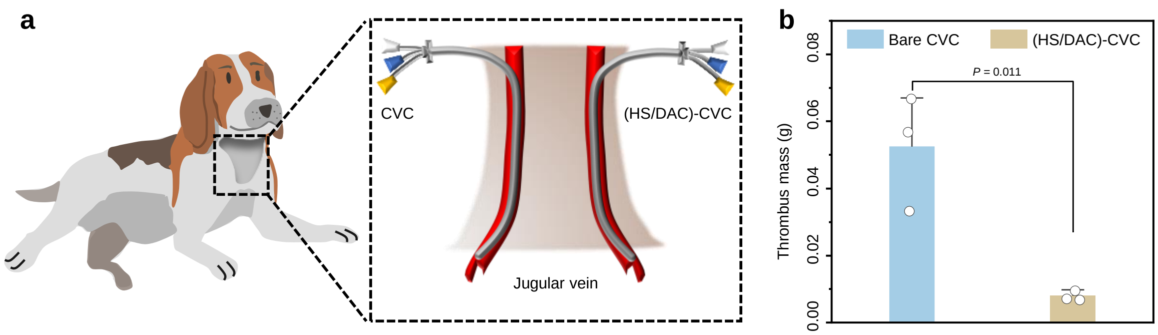

Supplementary Fig. 11 (a) Schematic of the acute canine model of HS/DAC coating in vivo. (b) Quality of thrombus formation on the surface of the catheter (mean ± SD, n = 3 independent samples). Two-sample Student's t test was used in b.

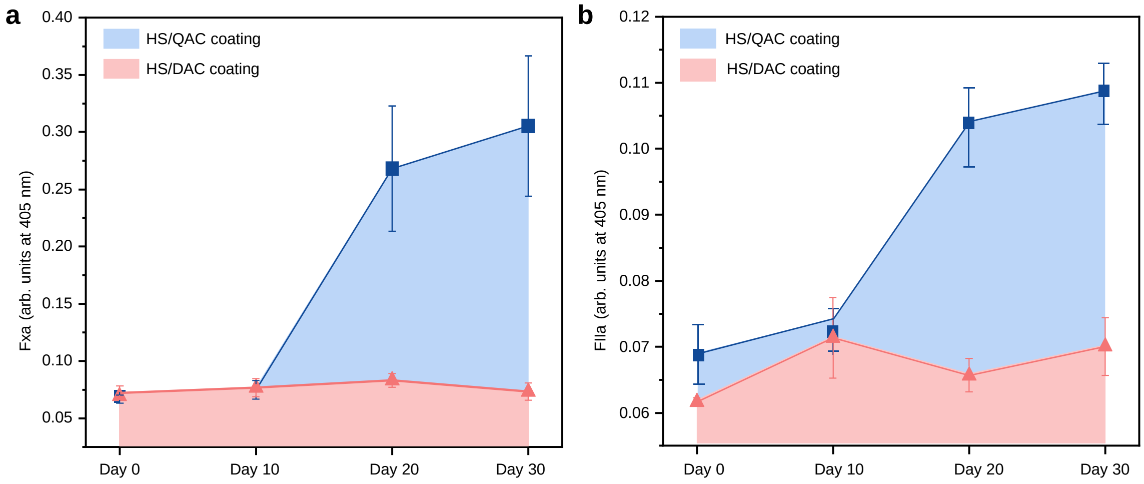

Supplementary Fig. 12 Anti-FXa (a) and anti-FIIa (b) assays of the coating after incubation in normal saline for 0, 10, 20 and 30 days (mean ± SD, n = 3 independent samples).

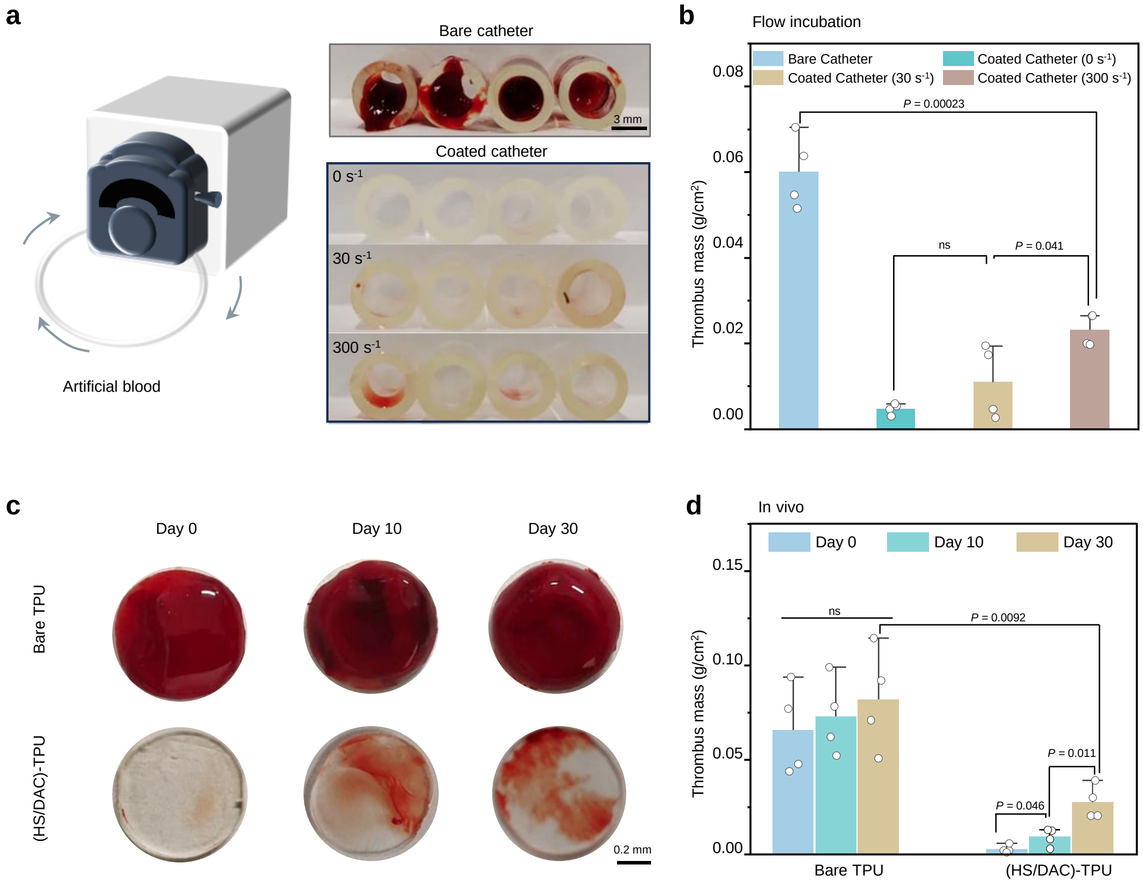

Supplementary Fig. 13 The long-term anti-thrombotic ability of HS/DAC coating. (a) Thrombosis on the surface of the HS/DAC-coated and bare catheter after flowing in artificial blood for 30 days. (b) The corresponding thrombus mass changes (mean ± SD, n = 4 independent samples). (c) Thrombosis on the surface of the HS/DAC-coated and bare surface after implanted in the rat for 0, 10 and 30 days. (d) The corresponding thrombus mass changes (mean ± SD, n = 3 independent samples). One-way ANOVA with Tukey's post hoc test was used in (b, d), ns: no significant difference. $P = 0.7$ in b and $P = 0.6$ in d.

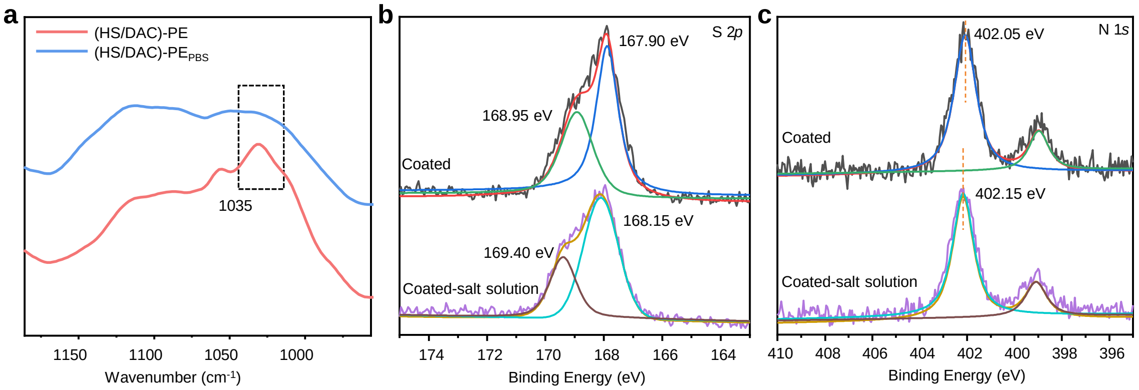

Supplementary Fig. 14 (a) ATR-FTIR spectra of sulfonic acid group in HS/DAC-coated TPU before and after treated with saline solution. XPS survey scan of (b) spectra of S 2*p* and (c) N 1*s* HS/DAC coating before and after treated with saline solution.

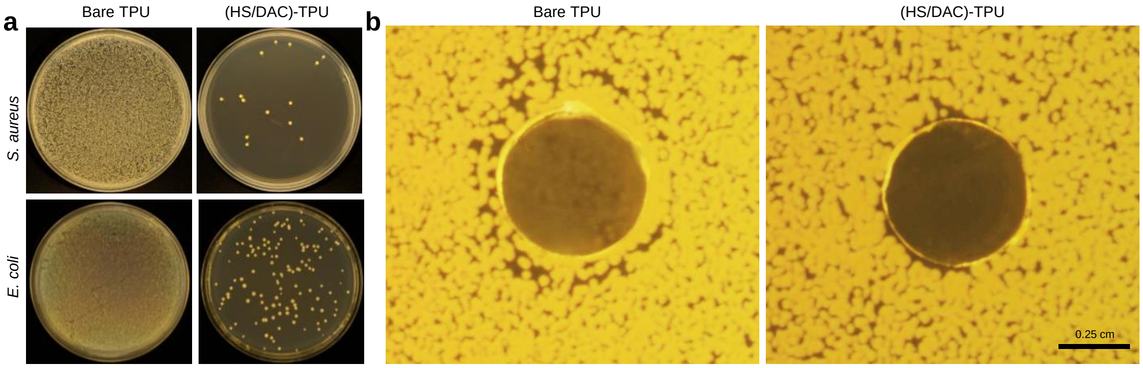

Supplementary Fig. 15 (a) Agar plate colony counting assay of bare and HS/DAC-coated TPU films. (b) Zone of inhibition tests of bare and HS/DAC-coated TPU films. Measurements were repeated three times independently with similar results.

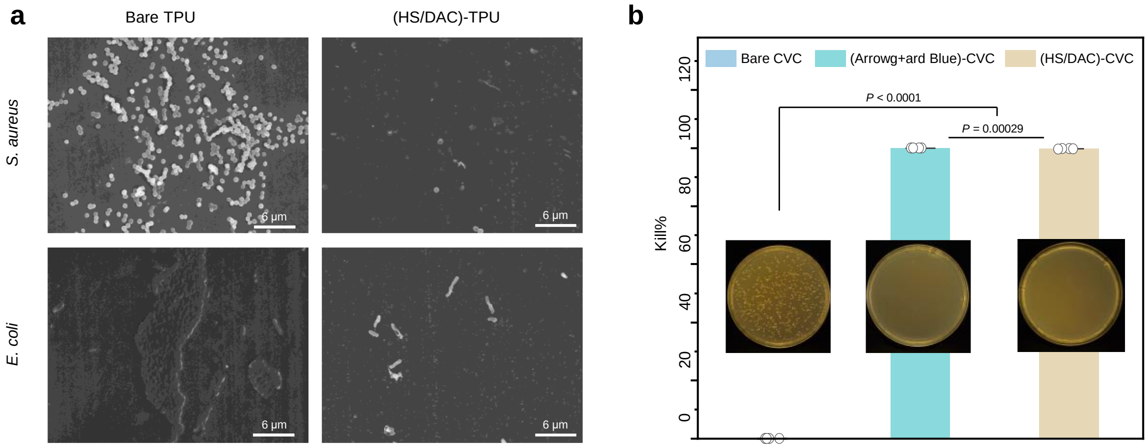

Supplementary Fig. 16 (a) SEM images of *S. aureus* and *E. coli* on the surface of bare TPU and HS/DAC-coated TPU films. Measurements were repeated three times independently with similar results. (b) The bactericidal rate of commercial (Arrowg + ard Blue)-CVC and (HS/DAC)-CVC (mean ± SD, n = 4 independent samples). One-way ANOVA with Tukey's post hoc test was used in b.

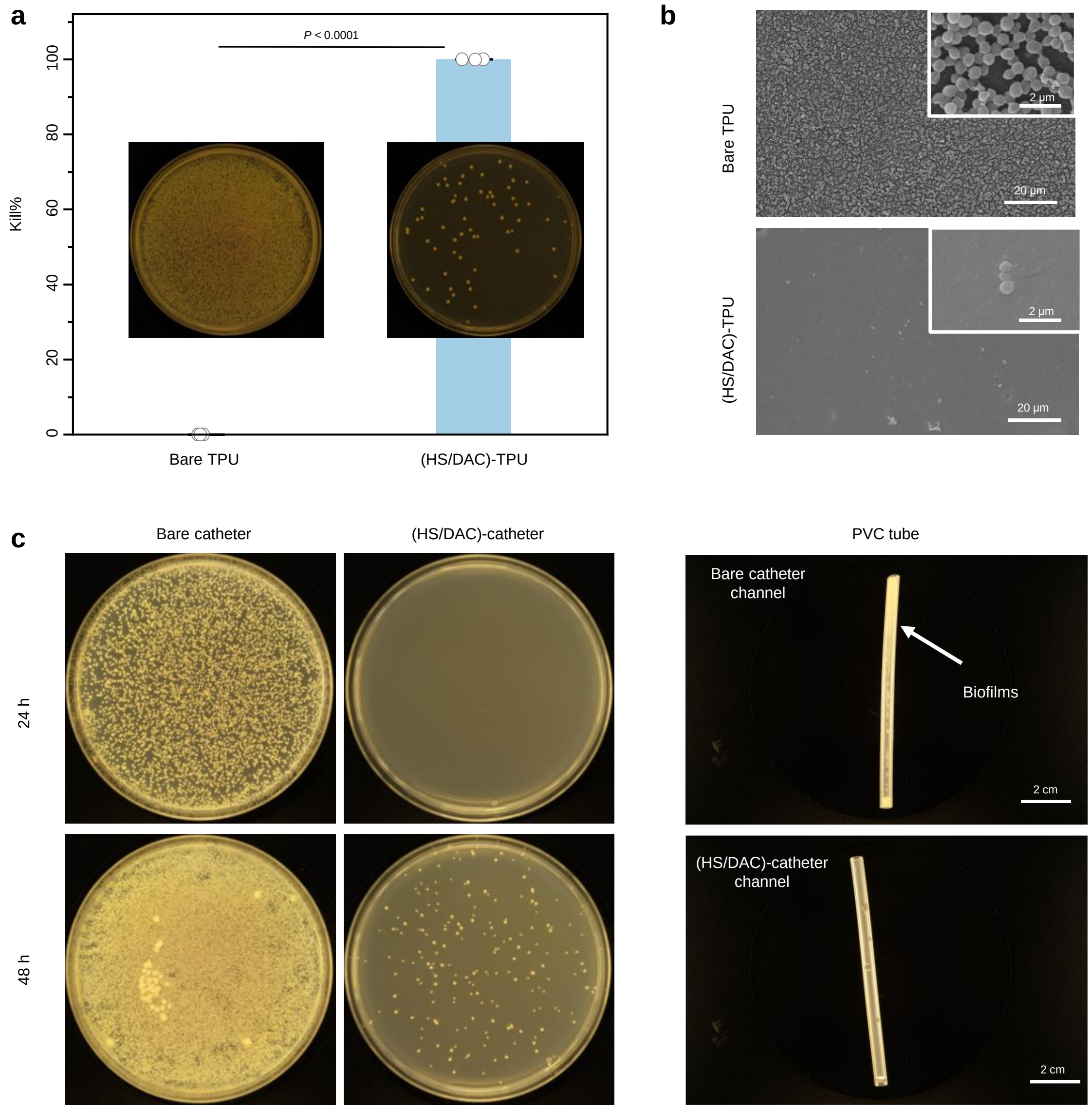

Supplementary Fig. 17 Antibiofilm activity of the HS/DAC coating. (a) Agar plate colony counting assay (mean ± SD, n = 3 independent samples) and (b) SEM images of the bare TPU and (HS/DAC)-TPU incubated with bacteria for 48 h. Measurements were repeated three times independently with similar results. (c) Images of catheters and live bacteria adhering to the inner surfaces of the catheters and PVC tubes in flow conditions at different times. Measurements were repeated three times independently with similar results. Two-sample Student's t test was used in a.

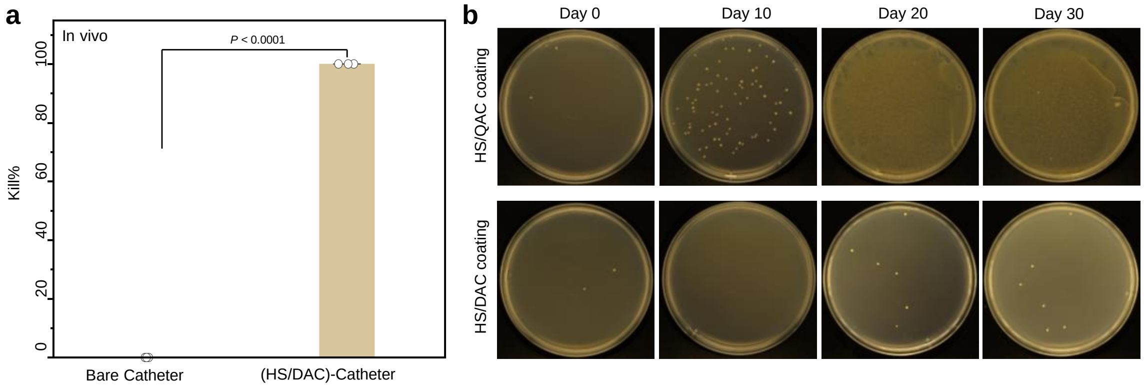

Supplementary Fig. 18 (a) Antibacterial rate of the HS/DAC-coated catheter in vivo (mean ± SD, n = 3 independent samples). (b)Images of live bacteria on the inner surface of the catheter with HS/QAC coating or HS/DAC coating shocked with 0.9% NaCl for 0, 10, 20 and 30 days.

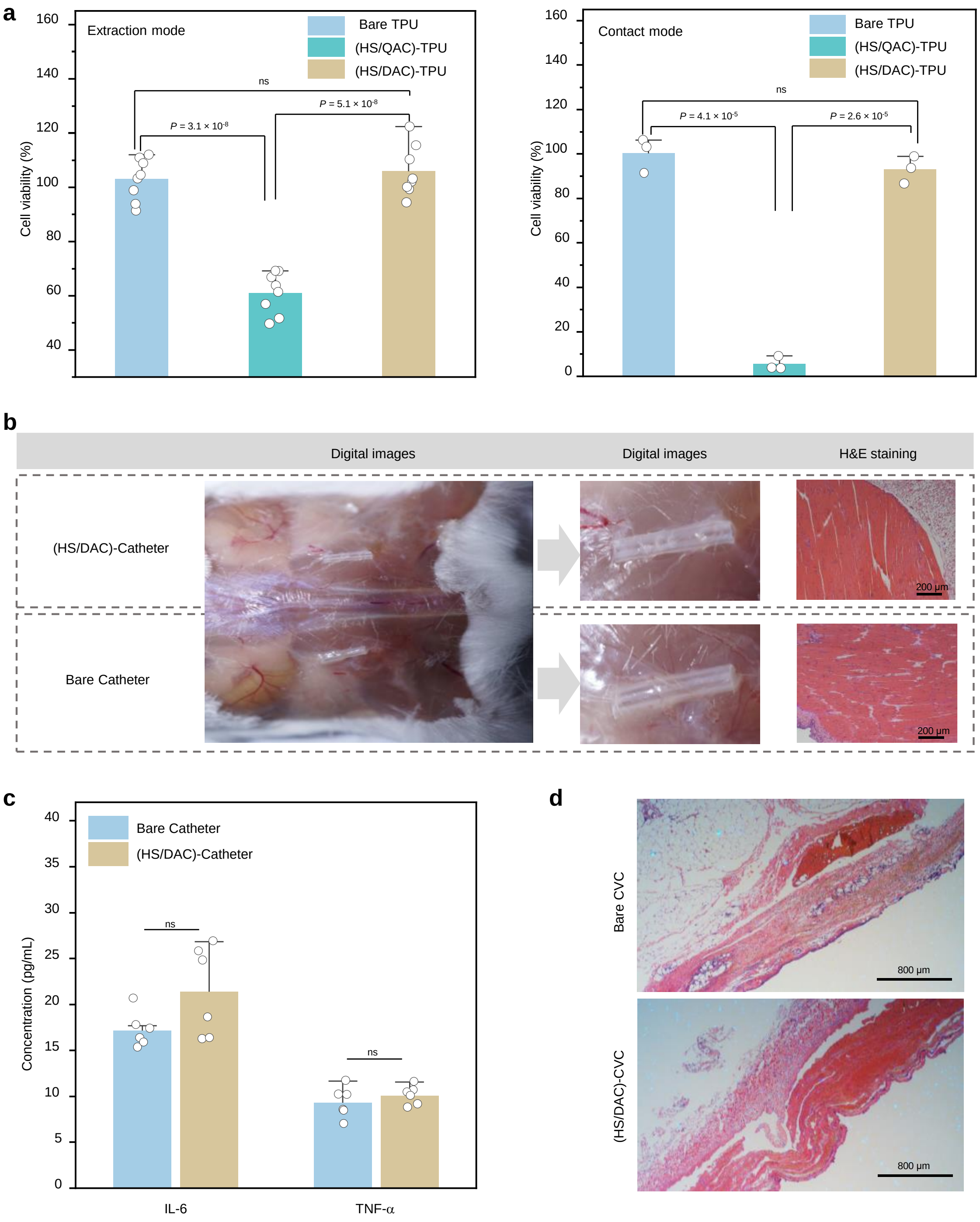

Supplementary Fig. 19 (a) Cell viability of bare, HS/QAC and HS/DAC-coated TPU via extraction mode (mean ± SD, n = 8 independent samples) and contact mode (mean ± SD, n = 3 independent samples). (b) Biocompatibility of the HS/DAC coating tested in vivo via H&E staining. Measurements were repeated three times independently with similar results. (c) Quantification of the cytokine in the tissue of the implantation position (mean ± SD, n = 6

independent samples). (d) H&E staining of canine vessel. Measurements were repeated three times independently with similar results. One-way ANOVA with Tukey's post hoc test was used in a. Two-sample Student's t test was used in c, ns: no significant difference. $P = 0.5$ in extraction mode and 0.3 in contact mode in b. $P = 0.7$ in IL-6 and 0.2 in TNF-α in c.

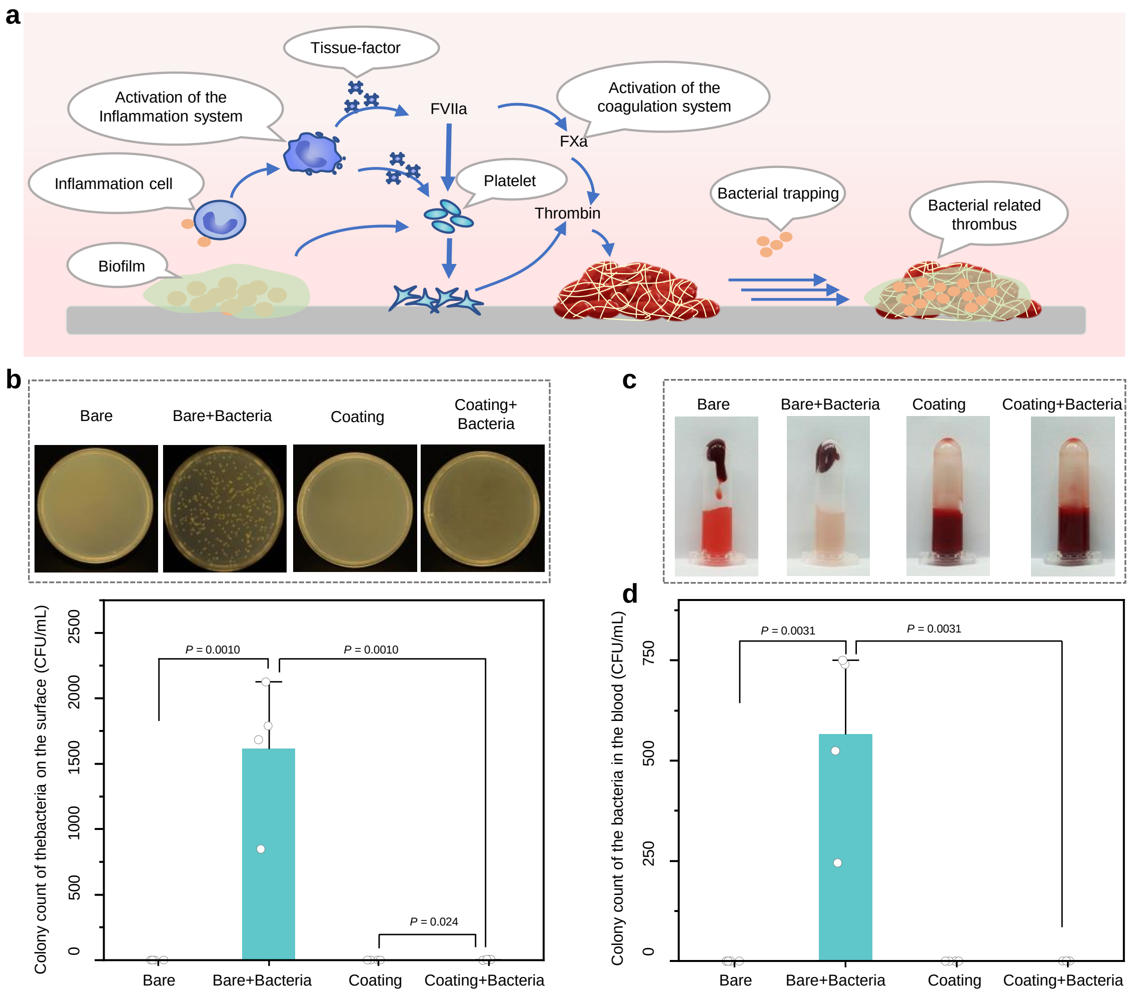

Supplementary Fig. 20 (a) Schematics of the bacterium-related thrombi formation mechanism on the infected catheters in vessels. (b) Images and agar plate colony count of alive bacteria on the surface (mean ± SD, n = 4 independent samples). (c) Images of thrombus and blood in circulating tubes. (d) Quantification of live bacteria in blood (mean ± SD, n = 4 independent samples). One-way ANOVA with Tukey's post hoc test was used in (b, d).

Supplementary Table 1

Table 1 Specific introduction to animal studies

Animal experiment	Strain	Age	Number (n)
Mice used in antibacterial study	BALB/C	6-8 weeks	9
Mice used in biocompatibility study	BALB/C	6-8 weeks	6
Rats used in antithrombotic stability study	Sprague-Dawley	7-8 weeks	4
Rats used in antibacterial stability study	Sprague-Dawley	7-8 weeks	4
Rabbits used in antithrombotic properties	New Zealand white rabbit	5 months	3
Rabbits used in bacteremia study	New Zealand white rabbit	5 months	4
Dogs used in the thrombogenicity study	Beagle	8 months	3

Supplementary Discussion

In Supplementary Fig. 2: Increasing the hydrophobic chain of DAC at the macroscopic level resulted in a gradual reduction in the required DAC concentration for the formation of white HS/DAC precipitation.

In Supplementary Fig. 3a: The FTIR spectra of the HS/DAC complex revealed characteristic absorption peaks of HS at 1640 cm^{-1}, 1220 cm^{-1}, and 1024 cm^{-1}, corresponding to -OH, C=O, S=O, and C-O-C bonds, respectively. Symmetric stretching of long alkyl chains (-CH_3) appeared at 2853 cm^{-1}, while an asymmetric angle change of CH_3 manifested at 1470 cm^{-1}. Additionally, vibration peaks of Si-O-C at 1078 cm^{-1} and 929 cm^{-1}, along with long-chain methylene at 720 cm^{-1}, were observed. In Supplementary Fig. 3b: Compared with the XPS spectrum of HS, new Si 2*p* peaks appeared in the spectrum of HS/DAC complexes, which means that complexes containing HS and DAC were constructed successfully by one-step assembly.

In Supplementary Fig. 4: Generally, hydrophobic polymeric materials like polyvinyl chloride (PVC) and polyurethane (PU) serve as the main components in IV catheters. Consequently, the liposoluble HS/DAC complex holds a distinct advantage in forming a uniform coating on inert surfaces, as these surfaces can be completely wetted in an organic solvent.

In Supplementary Fig. 5b: A contact-angle goniometer was applied to monitor the formation process of the coating according to the changes in wettability at the outermost layer of the coating. The surfaces changed from hydrophobic (98.57°) to hydrophilic (45.75°) after plasma treatment, indicating that hydroxylated surfaces were successfully achieved. When HS/DAC was coated on the surfaces, the water contact angle increased to 84.50° due to the hydrophobicity of the HS/DAC complex. The water contact angle increased again (96.51°) after a hydrolysis and condensation process, which may be attributed to the consumption of hydroxyl groups. In Supplementary Fig.5c, the (HS/DAC)-TPU coating demonstrated successful formation, as evidenced by the presence of S 2*p* peaks compared to bare TPU. In Supplementary Fig. 5d, the change in surface chemical structure a few micrometers from the interface were tested by FTIR. Compared with bare PE, the spectra of HS/DAC-coated PE exhibited chemical signatures unique to the HS/DAC complex, such as 3500 cm^{-1}, 1608 cm^{-1}, 1220 cm^{-1}, 1118 cm^{-1} and 932 cm^{-1}.

In Supplementary Fig. 9a: Clotting time was defined as the moment when no blood movement was observed upon tilting the glass bottles due to thrombus formation. The mean occlusion time for

uncoated glass bottles was 221 s, whereas the mean occlusion time for HS/DAC modified glass bottles exceeded 2 h.

In Supplementary Fig. 14: The sulfonic acid group's band at 1035 cm^{-1} significantly decreased and shifted, indicating the disruption of electrostatic interactions between the anion group and the quaternary amine group. XPS spectra revealed a shift in S 2*p* peaks from 167.9 and 168.95 eV for the HS/DAC coating to 168.15 and 169.40 eV for the salt-treated HS/DAC coating. Furthermore, the N 1*s* peak experienced a slight shift from 402.05 to 402.15 eV after salt treatment, confirming the dissociation between HS and DAC.

Supplementary References

1. Liu L, Shi H, Yu H, Zhou R, Yin J, Luan S. One-step hydrophobization of tannic acid for antibacterial coating on catheters to prevent catheter-associated infections. *Biomater. Sci.* **7**, 5035-5043 (2019).
2. Fan Y*, et al.* Immobilization of nano Cu-MOFs with polydopamine coating for adaptable gasotransmitter generation and copper ion delivery on cardiovascular stents. *Biomaterials* **204**, 36-45 (2019).
3. Zhang B*, et al.* Epigallocatechin gallate mediated sandwich-like coating for mimicking endothelium with sustained therapeutic nitric oxide generation and heparin release. *Biomaterials* **269**, 120418 (2021).
4. Maiti S, Misra JC. Non-Newtonian characteristics of peristaltic flow of blood in micro-vessels. *Commun Nonlinear Sci Numer Simul*. **18**, 1970-1988 (2013).
5. Kim S, Cho YI, Jeon AH, Hogenauer B, Kensey KR. A new method for blood viscosity measurement. *J Nonnewton Fluid Mech.* **94**, 47-56 (2000).
6. Wang X*, et al.* Temperature-responsive hierarchical polymer brushes switching from bactericidal to cell repellency. *ACS Appl. Mater. Interfaces.* **9**, 40930-40939 (2017).
7. Yu H*, et al.* Water-insoluble polymeric guanidine derivative and application in the preparation of antibacterial coating of catheter. *ACS Appl. Mater. Interfaces.* **10**, 39257-39267 (2018).
8. Yu H*, et al.* Fabrication of polylysine based antibacterial coating for catheters by facile electrostatic interaction. *Chem. Eng. J.* **360**, 1030-1041 (2019).
