# Supplementary material for: Heparin-network-mediated long-lasting coatings on intravascular catheters for adaptive antithrombosis and antibacterial infection
Source: Nat Commun. 2024 Jan 2;15:107. doi: 10.1038/s41467-023-44478-3 (PMC10761715; doi:10.1038/s41467-023-44478-3)
Supplement: Supplementary file 3 — Reporting Summary [file 41467_2023_44478_MOESM3_ESM.pdf]

nature portfolio

Corresponding author(s): Shifang Luan, Hengchong Shi

Last updated by author(s): Nov 28, 2023

Reporting Summary

Nature Portfolio wishes to improve the reproducibility of the work that we publish. This form provides structure for consistency and transparency in reporting. For further information on Nature Portfolio policies, see our Editorial Policies and the Editorial Policy Checklist.

Statistics

For all statistical analyses, confirm that the following items are present in the figure legend, table legend, main text, or Methods section.

n/a	Confirmed	
☐	☒	The exact sample size (*n*) for each experimental group/condition, given as a discrete number and unit of measurement
☐	☒	A statement on whether measurements were taken from distinct samples or whether the same sample was measured repeatedly
☐	☒	The statistical test(s) used AND whether they are one- or two-sided<br>*Only common tests should be described solely by name; describe more complex techniques in the Methods section.*
☒	☐	A description of all covariates tested
☒	☐	A description of any assumptions or corrections, such as tests of normality and adjustment for multiple comparisons
☐	☒	A full description of the statistical parameters including central tendency (e.g. means) or other basic estimates (e.g. regression coefficient) AND variation (e.g. standard deviation) or associated estimates of uncertainty (e.g. confidence intervals)
☐	☒	For null hypothesis testing, the test statistic (e.g. *F*, *t*, *r*) with confidence intervals, effect sizes, degrees of freedom and *P* value noted<br>*Give P values as exact values whenever suitable.*
☒	☐	For Bayesian analysis, information on the choice of priors and Markov chain Monte Carlo settings
☒	☐	For hierarchical and complex designs, identification of the appropriate level for tests and full reporting of outcomes
☒	☐	Estimates of effect sizes (e.g. Cohen's *d*, Pearson's *r*), indicating how they were calculated

Our web collection on statistics for biologists contains articles on many of the points above.

Software and code

Policy information about availability of computer code

Data collection	Water contact angle were performed on DSA100 drop-shape analyzer (KRÜSS, Hamburg, Germany);<br>Chemical structure were tested Attenuated total reflectance Fourier transform infrared spectroscopy (ATR-FTIR, BRUKER Vertex 70) and X-ray Photo electron Spectroscopy (XPS, VG Scientific ESCA MK II Thermo Avantage V 3.20 analyser).<br>Surface roughness were tested by Atomic Force Microscopy (AFM, Seiko, Japan).<br>The transmittance of the coating were tested by UV-Visible Spectrophotometer (Lambda 35, PerkinElmer, USA).<br>The stained bacteria and cells were observed by Zeiss Confocal LSM 700.<br>The morphology was observed by Model XL 30 ESEM FEG (SEM, Philips, Netherlands).
Data analysis	Statistical calculations were performed using Origin 2020 software (OriginLabs) and Microsoft Excel.Image J software (v1.6.0) was used to calculate the thrombus rate in lumen section. Digital microscopic images were analyzed by using Case Viewer (Version: 2.4.0.119028).

For manuscripts utilizing custom algorithms or software that are central to the research but not yet described in published literature, software must be made available to editors and reviewers. We strongly encourage code deposition in a community repository (e.g. GitHub). See the Nature Portfolio guidelines for submitting code & software for further information.

Data

Policy information about availability of data

All manuscripts must include a data availability statement. This statement should provide the following information, where applicable:

- Accession codes, unique identifiers, or web links for publicly available datasets
- A description of any restrictions on data availability
- For clinical datasets or third party data, please ensure that the statement adheres to our policy

All data are available in the main text, Supplementary Information, or Source Data file. Source data are provided with this paper. If any raw data files are needed in another format, they are available from the corresponding author upon request.

Research involving human participants, their data, or biological material

Policy information about studies with human participants or human data. See also policy information about sex, gender (identity/presentation), and sexual orientation and race, ethnicity and racism.

Reporting on sex and gender	This study did not involve human
Reporting on race, ethnicity, or other socially relevant groupings	This study did not involve human
Population characteristics	This study did not involve human
Recruitment	This study did not involve human
Ethics oversight	This study did not involve human

Note that full information on the approval of the study protocol must also be provided in the manuscript.

Field-specific reporting

Please select the one below that is the best fit for your research. If you are not sure, read the appropriate sections before making your selection.

☒ Life sciences ☐ Behavioural & social sciences ☐ Ecological, evolutionary & environmental sciences

For a reference copy of the document with all sections, see nature.com/documents/nr-reporting-summary-flat.pdf

Life sciences study design

All studies must disclose on these points even when the disclosure is negative.

Sample size	No statistical method was used to predetermine the sample size for each study. The appropriate sample size was used based on the published literatures on similar evaluations (such as Nature 556, 103–107 (2018)). For in vitro studies,the appropriate sample size (n=3-8) was used. For in vitro studies,the appropriate sample size (n=3-9) was used for ex vivo experiments. For in vivo studies, each group contains 3-4 for evaluating the statistical significance. Mice used in antibacterial study (n=9); Mice used in biocompatibility study (n=6); Rats used in antithrombotic stability study (n=4); Rats used in antibacterial stability study (n=4); Rabbits used in antithrombotic properties (n=3); Rabbits used in bacteremia study (n=4); Dogs used in the thrombogenicity study (n=3). A precise value of 'n' were provided in the legends of figures. Samples were randomly selected for testing. Sample size was chosen to ensure reproducibility of the experiments in accordance with the replacement, reduction and refinement principles of animal ethics regulation.
Data exclusions	No data was excluded in this study.
Replication	All experiments were performed with independent replicates. At least three independent samples were performed for each experiment. All replications were successful.
Randomization	All samples were randomly allocated into experimental groups
Blinding	The operator was blinded to group allocation during H&E staining. Blinding was not relevant to other work since the metrics were quantified and objectively analyzed.

Reporting for specific materials, systems and methods

We require information from authors about some types of materials, experimental systems and methods used in many studies. Here, indicate whether each material, system or method listed is relevant to your study. If you are not sure if a list item applies to your research, read the appropriate section before selecting a response.

Materials & experimental systems

n/a	Involved in the study	
☐	☒	Antibodies
☐	☒	Eukaryotic cell lines
☒	☐	Palaeontology and archaeology
☐	☒	Animals and other organisms
☒	☐	Clinical data
☒	☐	Dual use research of concern
☒	☐	Plants

Methods

n/a	Involved in the study	
☒	☐	ChIP-seq
☒	☐	Flow cytometry
☒	☐	MRI-based neuroimaging

Antibodies

Antibodies used	Anti-fibrinogen gamma chain antibody; Anti-CD14; Anti-CD3;Alexa Fluor 555-labeled Donkey Anti-Rabbit IgG(H+L) antibody and Alexa Fluor 488-labeled Goat Anti-Rabbit IgG(H+L) antibody
Validation	There is no novel antibodies in this study. All antibodies used were well described in literature or in the manufacturer's protocols. The protocols for antibody dilution and incubation time in the immunofluorescence assay were optimized. Produced recombinantly (animal-free) for high batch-to-batch consistency and long term security of supply. Anti-fibrinogen gamma chain antibody, mouse monoclonal [5A6] (ab119948, 1:1000), anti-CD14 antibody, rabbit monoclonal [EPR21847] (ab221678, 1:1000), anti-CD3 antibody, rabbit monoclonal [SP162] (ab135372, 1:1000) was purchased from Abcam. Alexa Fluor 555-labeled Donkey Anti-Rabbit IgG(H+L) antibody (A0453, 1:200) and Alexa Fluor 488-labeled Goat Anti-Rabbit IgG(H+L) antibody (A0423, 1:200) were purchased from Beyotime Biotechnology (Shanghai, China).

Eukaryotic cell lines

Policy information about cell lines and Sex and Gender in Research

Cell line source(s)	In this study, the L929 murine fibroblast cell line (FH0534) was purchased from FuHeng Biology (Shanghai, China).
Authentication	L929 cells were authenticated by FuHeng based on STR profiling technology.
Mycoplasma contamination	The cell lines were not additionally validated in this study. The cell lines were tested negative for mycoplasma contamination when they came out of the library.
Commonly misidentified lines (See ICLAC register)	No commonly misidentified cell lines were used in the study

Animals and other research organisms

Policy information about studies involving animals; ARRIVE guidelines recommended for reporting animal research, and Sex and Gender in Research

Laboratory animals	Female BALB/c mice (6-8 weeks old) , female Sprague-Dawley rats (SD rats, 160-200g, 7-8 weeks old) and female New zealand white rabbits (3.0-3.5 kg, 5 months), female canines (Beagle, 12-14 kg, 8 months) were used for our experiments.<br>Animals were housed at an ambient temperature of 25 °C (24-26 °C), humidity of 30 % and a photoperiod of 12 h light/12 h dark. In addition, a standard diet and water were given.
Wild animals	This study did not involve wild animals.
Reporting on sex	All female animals were used in this study, and gender differences were not considered. The evaluation of medical devices in our present work mainly focused on the antithrombotic and antibacterial tests. In these experiments, gender has a relatively little impact on the antithrombotic and antibacterial results of medical devices. Preference was given to female vertebrate that were gentler in the rearing process.
Field-collected samples	The study did not involve samples collected from the field.
Ethics oversight	All animal procedures were performed in accordance with the Guidelines for the Care and Use of Laboratory Animals of the Chinese Academy of Sciences and approved by the Animal Ethics Committee of Changchun Institute of Applied Chemistry, Chinese Academy of Sciences (no. 20210027).

Note that full information on the approval of the study protocol must also be provided in the manuscript.
